# Synthesis Characterization and DNA Interaction Studies of a New Zn(II) Complex Containing Different Dinitrogen Aromatic Ligands

**DOI:** 10.1155/2012/571913

**Published:** 2012-08-26

**Authors:** Nahid Shahabadi, Somaye Mohammadi

**Affiliations:** Department of Inorganic Chemistry, Faculty of Chemistry, Razi University, Kermanshah 74155, Iran

## Abstract

A mononuclear complex of Zn(II), [Zn(DIP)_2_ (DMP)] (NO_3_)_2_ *·*2H_2_O in which DIP is 4,7-diphenyl-1,10-phenanthroline and DMP is 4,4′-dimethyl-2,2′-bipyridine has been prepared and characterized by ^1^HNMR spectroscopy, FT-IR, UV-Vis and elemental analysis techniques. DNA-binding properties of the complex were studied using UV-vis spectra, circular dichroism (CD) spectra, fluorescence, cyclic voltammetry (CV), and viscosity measurements. The results indicate that this zinc(II) complex can intercalate into the stacked base pairs of DNA and compete with the strong intercalator ethidium bromide for the intercalative binding sites.

## 1. Introduction

 In recent years, many researches [1–3] have been focused on interaction of small molecules with DNA. DNA is generally the primary intracellular target of anticancer drugs, so the interaction between small molecules and DNA can cause DNA damage in cancer cells, blocking the division of cancer cells and resulting in cell death [4, 5]. Small molecule can interact with DNA through the following three noncovalent modes: intercalation, groove binding, and external electrostatic effects. Among these interactions, intercalation is one of the most important DNA-binding modes, which is related to the antitumor activity of the compound. In this regard, mixed-ligand metal complexes were found to be particularly useful because of their potential to bind DNA *via *a multitude of interactions and to cleave the duplex by virtue of their intrinsic chemical, electrochemical, and photochemical reactivities [6, 7]. It has been reported that the intercalating ability of the complex was involved in the planarity of ligands, the coordination geometry, ligand donor atom type, and the metal ion type. Additionally, metal complexes with tris-dinitrogen ligands and their analogs have attracted much attention for the chiral recognition of DNA double helices with the enantiomeric complexes and for the photochemical electron transfer reaction initiated by the complex bound to DNA [8–10]. A singular advantage in the use of these metallointercalators for such studies is that the ligands or the metal ion in them can be varied in an easily controlled manner to facilitate individual applications [11–13]. In this regards, binding of zinc(II) complexes to DNA has attracted much attention [14]. In this study, we investigated the mode of DNA binding of a new zinc(II) complex containing mix aromatic dinitrogen ligands. Many techniques have been used to investigate the interactions of complex with DNA. These include (i) molecular spectroscopy methods such as UV spectrophotometry [15], fluorescence [16], cyclic voltametry (CV), and circular dichroism (CD) spectropolarimetry [17] and (ii) dynamic viscosity measurements [18].

## 2. Experimental

 All chemicals such as Zn(NO_3_)_2_·6H_2_O, 4,7-diphenyl-1,10-phenanthroline (DIP), and 4,7-dimethyl-1,10-phenanthroline (DMP) were purchased from Merck, and Tris-HCl highly polymerized calf thymus DNA (CT-DNA) were purchased from Sigma Co. Experiments were carried out in Tris-HCl buffer at pH = 7.0. A solution of calf thymus DNA gave a ratio of UV absorbance at 260 and 280 nm more than 1.8, indicating that DNA was sufficiently free from protein [19]. The stock solution of CT-DNA was prepared by dissolving of DNA in 10 mM of the Tris-HCl buffer at pH = 7.0. The DNA concentration (monomer units) of the stock solution (1 × 10^−2^ M per nucleotide) was determined by UV spectrophotometer, in properly diluted samples, using the molar absorption coefficient 6600 M^−1 ^cm^−1^ at 258 nm. The stock solutions were stored at 4°C and used over no more than 4 days. 

### 2.1. Synthesis of the [Zn(DIP)_2_(DMP)](NO_3_)_2_·2H_2_O Complex

**Figure 1:**
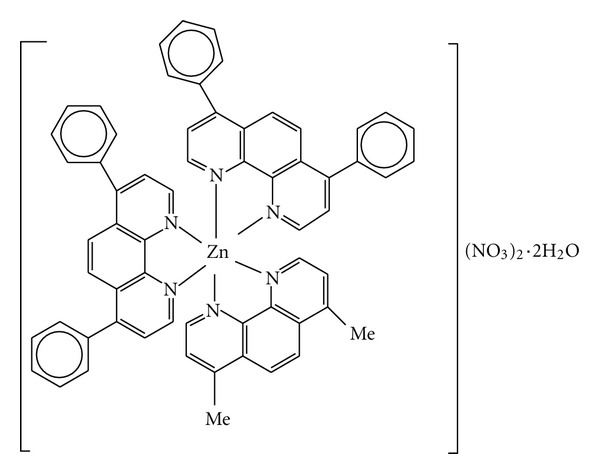
The structure of [Zn(DIP)_2_(DMP)](NO_3_)_2_·H_2_O complex.

The complex [Zn(DIP)_2_(DMP)](NO_3_)_2_·2H_2_O (Figure 1) was prepared by mixing the appropriate molar quantities of the ligands and the metal salt using the following procedure. A methanolic solution of Zn(NO_3_)_2_·6H_2_O (0.297 g, 1 mmol) was stirred with a methanolic solution (4 mL) of the 4,7-dimethyl-1,10-phenanthroline (DMP) (0.21 g, 1 mmol) for *ca*. 1 h. To the above mixture, a methanolic solution (5 mL) of 4,7-diphenyl-1,10-phenanthroline (0.664 g, 0.2 mmol) was added in a 1 : 1 : 2 molar ratio, and the stirring was continued for *ca*. 1 h. The obtained solid product was filtered and washed with methanol. A bright pale brown precipitate was formed, which was filtered off and washed with ice-cold water and diethyl ether. Yield: 63%. Elemental analysis for ZnC_62_H_44_N_6_(NO_3_)_2_·2H_2_O. Found (calculated): C, 65.8 (67.82); H, 4.0 (4.37); N, 11.0 (10.21). IR data (cm^−1^): 1545 (ring), 1515 (C=C), 1429 (CCH), 840 (Phen), 729 (Phen), 1342 (Me), 486 (Zn–N); molar conductance, (Ω^−1^ cm^2^ mol^−1^) in DMF: 121 (1 : 2 electrolyte). In the ^1^H-NMR spectra of the Zn(II) complexes, the protons of the ligands are shifted downfield due to coordination to the metal ion.

## 3. Instrumentation

^1^HNMR spectra were recorded using a Bruker Avance DPX200 MHz (4.7 Tesla) spectrometer with CDCl_3_ as the solvent. The elemental analysis was performed using a Heraeus CHN elemental analyzer. Absorbance spectra were recorded using an HP spectrophotometer (Agilent 8453) equipped with a thermostated bath (Huber Polystat cc1). Absorption titration experiments were conducted by keeping the concentration of complex constant (2.9 × 10^−5 ^M) while varying the DNA concentration from 0 to 1.9 × 10^−4 ^M (*r*_*i*_ = [DNA]/[complex] = 0.0, 0.2, 0.5, 0.75, 1.5, 2, 3, 4). Absorbance values were recorded after each successive addition of DNA solution and equilibration (ca. 10 min). The data were then fitted to (1) to obtain intrinsic binding constant, *K*_*b*_ [20]:

(1) $$\begin{array}{c} \frac{\left[\text{DNA}\right]}{\left(\varepsilon_{a}-\varepsilon_{f}\right)}=\frac{\left[\text{DNA}\right]}{\left(\varepsilon_{b}-\varepsilon_{f}\right)}+\frac{1}{K_{b}\left(\varepsilon_{b}-\varepsilon_{f}\right)}, \end{array}$$

where *ε*_*a*_, *ε*_*f*_, and *ε*_*b*_ are the apparent, free, and bound complex extinction coefficients, respectively. In particular, *ε*_*f*_ was determined by a calibration curve of the isolated metal complex in aqueous solution; following Beer's law, *ε*_*a*_ was determined as the ratio between the measured absorbance and the Zn(II) complex concentration, A_obs_/[complex]. A plot of [DNA]/(*ε*_*a*_ − *ε*_*f*_) versus [DNA] gave a slope of 1/(*ε*_*b*_ − *ε*_*f*_) and a *Y* intercept equal to 1/*K*_*b*_(*ε*_*b*_ − *ε*_*f*_); *K*_*b*_ is the ratio of the slope to the *Y* intercept. 

CD measurements were recorded on a JASCO (J-810) spectropolarimeter, keeping the concentration of DNA constant (5.9 × 10^−5 ^M) while varying the complex concentration (*r*_*i*_ = [complex]/[DNA] = *r*_*i*_ = 0, 0.4, 0.5).

 Viscosity measurements were made using a viscosimeter (SCHOT AVS 450) maintained at 25.0 ± 0.5°C using a constant temperature bath. The DNA concentration was fixed at 5.9 × 10^−5 ^M, and flow time was measured with a digital stopwatch. The mean values of three measurements were used to evaluate the viscosity g of the samples. The values for relative specific viscosity (*η*/*η*_0_)^1/3^, where *g*_0_ and *g* are the specific viscosity contributions of DNA in the absence (*η*_0_) and in the presence of the complex (*η*), were plotted against *r*_*i*_ (*r*_*i*_ = [DNA]/[complex] = 0.0, 0.3, 0.6, 0.9, 1.2, 1.5, 2.0). 

 All fluorescence measurements were carried out with a JASCO spectrofluorimeter (FP6200) by keeping concentration of complex constant while varying the DNA concentration from 0 to 36.9 × 10^−5^ (*r*_*i*_ = [DNA]/[complex] = 0.0, 0.2, 0.4, 0.6, 0.8, 1) at three different temperatures (298, 308, 318 K).

 The cyclic voltammetric, linear sweep voltammetry, and differentials pulse voltammetry (DPV) measurements were performed using an AUTOLAB model (PG STAT C), with a three-electrode system: a 0.10 cm diameter Glassy carbon (GC) disc as working electrode, an Ag/AgCl electrode as reference electrode, and a Pt wire as counter electrode. Electrochemical experiments were carried out in a 25 mL voltammetric cell at room temperature. All potentials are referred to the Ag/AgCl reference. Their surfaces were freshly polished with 0.05 mm alumina prior to each experiment and were rinsed using double-distilled water between each polishing step. The supporting electrolyte was 0.01 M of Tris-HCl buffer solution (pH = 7.4) which was prepared with double-distilled water. Before experiments, the solution was deaerated via purging with pure nitrogen gas for 1 min, and during measurements a stream of nitrogen was passed over the solution. The current-potential curves and experimental data were recorded on software GPES [21].

## 4. Results and Discussion

### 4.1. Synthesis and Characterization of [Zn(DIP)_2_(DMP)](NO_3_)_2_·2H_2_O Complex

**Figure 2:**
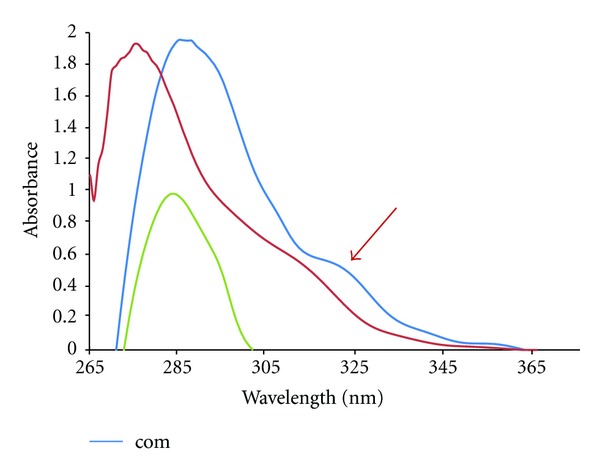
The UV-Vis spectra of the free ligands (DIP and DMP) and the zinc complex.

The complex conforms to the formula [Zn(DIP)_2_(DMP)](NO_3_)_2_·2H_2_O determined on the basis of elemental analysis. The IR spectrum of the complex was characterized by the appearance of a band at 486 cm^−1^ due to the *ν*(Zn–N). The coordination of the nitrogen atoms is confirmed with the presence of this band. However, the broad band at 3400–3500 cm^−1^ is assigned to [*ν*(H_2_O)]. ^1^ *HNMR*; in the aromatic region the signals at **δ** = 8.8, **δ** = 7.6, **δ** = 7.4, and **δ** = 7.2 were assigned to the protons of DIP ligand. The protons of DIP ligands are seen to be shifted (*≈* −0.2 ppm) with corresponding free ligand and suggesting complexation. The UV-Vis spectra of the complex and the free ligands were recorded. The spectrum of the complex shows a d-d band at around 365 nm. This band may be assign to 2Eg to 2T_2 _g transitions, characteristics for a distorted octahedral structure. In addition, the intense higher energy bands at around 286 and 288 nm can be attributed to intraligand *π*-π* transitions which shifted after complexation (Figure 2). Based on the elemental analysis, electronic, IR, and ^1^HNMR data, the proposed structures of the complex are given in Figure 1.

### 4.2. DNA Interaction Studies

#### 4.2.1. UV-Vis Spectroscopy

**Figure 3:**
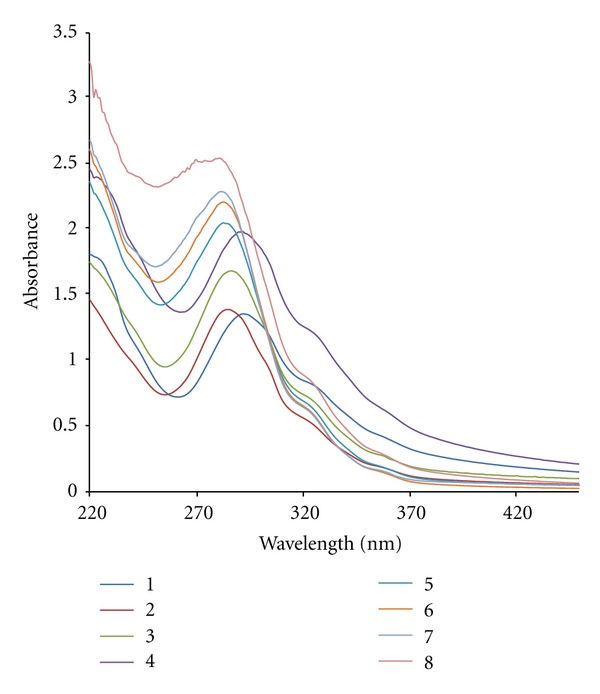
Absorption spectra of Zn(II) complex (2.9 × 10^−5 ^M) in the absence and presence of increasing amounts of CT-DNA: *r*_*i*_ = 0.0, 0.2, 0.5, 0.75, 1.5, 2, 3, 4.

 Absorption spectroscopy is one of the most useful techniques to study the binding of any drug to DNA. The extent of hypochromism generally indicates the intercalative binding strength [22]. “Hyperchromic” and “hypochromic” effects are the spectra features of DNA concerning its double helical structure [23]. Hyperchromism has been observed for the interaction of many drugs with DNA [24]. The hyperchromic effect might be ascribed to external contact (electrostatic binding [25]) or to partial uncoiling of the helix structure of DNA, exposing more bases of the DNA [26].  Figure 3 illustrates the spectral changes in aqueous solution for the Zn(II) complex during titration with increasing amounts of CT-DNA at *r*_*i*_ values of 0, 0.05, 0.5, 1.5, 5. The initial spectrum is of a fixed concentration (2.9 × 10^−5^ M^−1^) of the free complex in the absence of CT-DNA. In the UV region, the intense absorption band at 290 nm is attributed to the interligand *π*-π* transitions of the coordinated groups. The appreciable changes in the position of the maximum wavelength are observed on addition of CT-DNA (7 nm; blue shift), and the intensity of the band of the Zn(II) complex at 290 nm increases, resulting in hyperchromism. The hyperchromic effect may also be due to the electrostatic interaction between positively charged [Zn(DIP)_2_(DMP)]^2+^ complex unit and the negatively charged phosphate backbone at the periphery of the double-helix CT-DNA [27]. 

Structurally, intercalation to DNA may not be one of the binding modes, since the tris(bidentate) ligand strands wrap around the zinc center, possessing pseudo-threefold rotation axis that passes through the metal ions. Therefore, the complete intercalation of the ligands between a set of adjacent base pairs is sterically impossible, but some partial intercalation can be envisioned [28]. It should be pointed out that the absorbance of the shoulder at 326 nm was not changed upon addition of DNA, suggesting that metal-ligand supramolecular architecture was not significantly modified by binding. 

The intrinsic binding constant, *K*_*b*_, of the [Zn(DIP)_2_(DMP)]^2+^ complex was determined to be (3.1 ± 0.02) × 10^6^ M^−1^ using (1). Interestingly, the *K*_*b*_ values obtained for our Zn(II) complex sample are very much higher than those for the other known ordinary complexes containing 1,10-phenanthroline and/or modified phenanthroline ligands available in the literature [28]. This shows that comparatively our complex samples can bind very strongly with DNA. Viscosity and circular dichroism measurements could have been helpful to us to confirm the intercalative behavior.

#### 4.2.2. Fluorescence Spectroscopy

**Figure 4:**
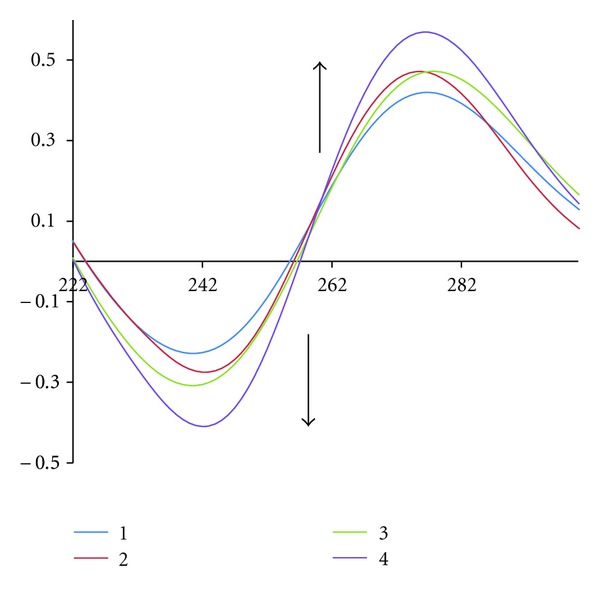
Circular dichroism spectra of CT-DNA (5.9 × 10^−5 ^M) in Tris buffer (10 mM), in the presence of increasing amounts of the complex.

 As the zinc complex is luminescent in the absence of DNA, it does show appreciable increase in emission upon addition of CT-DNA (Figure 4). This figure shows that a regular increase in the fluorescence intensity of the complex (1) with a shift of fluorescence emission maximum (414–416 nm) took place upon increasing the concentration of DNA at 25.0°C and at pH = 7.2. These fluorescence enhancements indicate that the complex interacted with DNA and the quantum efficiency of complex was increased. Like the quenching process, the enhancement constant can be obtained by the following [29]:

(2) $$\begin{array}{c} \frac{F_{0}}{F}=1-K_{E}\left[E\right]. \end{array}$$

If a dynamic process is part of the enhancing mechanism, the above equation can be written as follows:

(3) $$\begin{array}{c} \frac{F_{0}}{F}=1-k_{D}\left[E\right]=1-K_{B}\tau_{0}, \end{array}$$

where *k*_*D*_ is the dynamic enhancement constant (like a dynamic quenching constant), *k*_*B*_ is the bimolecular enhancement constant (like to a bimolecular quenching constant), and *τ*_0_ is the lifetime of the fluorophore in the absence of the enhancer. The dynamic enhancement constants of the complex at different temperatures were calculated using (3) (Figure 4, Table 1). Since fluorescence lifetimes are typically near 10^−8^ s, the bimolecular enhancement constant (*k*_*B*_) was calculated from *k*_*D*_ = *k*_*B*_ *τ*_0_ (Table 1).

By considering the equivalency of the bimolecular quenching and enhancement constants, it can be seen that the latter is greater than the largest possible value (1.0 × 10^10^ M^−1^s^−1^) in aqueous medium. Thus, the fluorescence enhancement is not initiated by a dynamic process; it is suggested that a static process involves complex formation in the ground state [30].

#### 4.2.3. Equilibrium Binding Titration

 The binding constant (*K*_*f*_) and the binding stoichiometry (*n*) for the complex formation between the complex and DNA were measured using the following [30]:

(4) $$\begin{array}{c} \frac{\log\left(F-F_{0}\right)}{F}=\log K_{f}+n\log\left[\text{DNA}\right]. \end{array}$$

Here *F*_0_ and *F* are the fluorescence intensities of the fluorophore in the absence and in the presence of different concentrations of DNA, respectively. The linear equations of log (*F* − *F*_0_)/*F* versus log [DNA] at different temperature are shown in Table 1. The values of *K*_*f*_ clearly underscore the remarkably high affinity of the complex to DNA.

#### 4.2.4. Thermodynamic Parameters of DNA Binding

 To have a better understanding of thermodynamics of the reaction between the complex and DNA, it is useful to determine the contributions of enthalpy and entropy of the reaction. Therefore, the evaluation of formation constant for the complex-DNA at three different temperatures (298, 308, 318 K) allows determining the thermodynamic parameters such as enthalpy (Δ*H*) and entropy (Δ*S*) of complex-DNA formation by van't Hoff's equation and plotting log *K*_*f*_ versus 1/*T*. The positive slope of the plot (−Δ*H*/*R*, *R* is the gas constant) indicates that the reaction of DNA with the complex is exothermic and enthalpy favored. The Δ*H* and Δ*S* values of the complex-DNA were −179.948 ± 0.6 kJ/mol and −488.042 ± 2 J/mol K, respectively. In general, electrostatic interactions exhibit small enthalpy and positive entropy changes. Hydrophobic interactions are generally indicated by positive enthalpy and entropy changes. Hydrogen bonding and van der Waals interactions are usually characterized by negative standard enthalpies of interaction [30, 31]. From the thermodynamic data, it is quite clear that while complex formation is enthalpy favored, it is also entropy disfavored. Formation of the complex therefore results in a more ordered state, possibly due to the freezing (fixing) of the motional freedom of both the complex and DNA molecules. These thermodynamic data are lower than those of previously reported for an intercalator [32] and confirmed the non-intercalating interaction mode.

The analogous [Ru(phen)_3_]^2+^ [33, 34] which is suggested to bind electrostatically to DNA has been reported to exhibit a positive enthalpy change, but Mudasir et al. [35] found that the mode of binding of CT-DNA with [Fe(phen)(DIP)_2_]^2+^ (Δ*H* = −33.1 kJ mol^−1^) was intercalation. Furthermore, Chaires studies of the thermodynamic parameters of drug-DNA interactions revealed that for all the intercalators except actinomycin, the binding enthalpy changes are large and negative [36]. When cationic binding agent such as this complex binds to DNA, it is likely replace a countercation from the compact inner (stern) layer or the defuse outer layer surrounding DNA. This counter ion release process is believed to be nearly entirely entropic [37] and the signs of Δ*H* and Δ*S* obtained for DNA binding of complex should be positive. Since the complex is also a cationic binding agent and its structure is similar to other complexes which were previously studied [35], it is expected that counter ion release, hydration, and hydrophobic interaction should occur in this complex. Evidently, the overall negative enthalpy and entropy changes are obtained for DNA binding of this complex. In addition to counter ion release, there should be another type of molecular interaction process in the DNA binding of the complex from which large negative enthalpy and entropy changes are produced. Intercalation of the phenyl group and partial phenanthroline ring of one DIP ligand in complex which involves *π*-stacking interaction between these planar substituents and DNA base pairs seems to be consistent with the enthalpy and entropy changes. Intercalation, in which two phenyl rings of the ligand are inserted into the DNA base pairs, may be less favorable, because the sheer size of the DIP ligand would preclude simultaneous intercalation of both phenyl rings as the diameter of the ligand is much larger than the width of base pairs. In addition, the crystal structure data of solid [Cu(bcp)_2_]BF_4_ (bcp = 2,9-dimethyl-4,7-diphenyl-1,10-phenanthroline) [38] complexes show that all the phenyl groups are skewed about 40 out of phenanthroline plane owing to steric effect between hydrogen atoms in the phenyl and phenanthroline rings. Intercalation of the entire DIP moiety thus would require that the phenyl groups rotate into the plane of the phenanthroline moiety in order to minimize stacking. This may produce unfavorable intermolecular interaction between the complex and DNA. In order to explain such steric hindrance, a model of partial intercalation of the phenanthroline ligand with only one phenyl substituent and partial phen ring inserted between the base pairs in the major groove (an asymmetric docking) has been proposed [39]. In this model, the other phenyl group and nonintercalating DIP ligands are aligned along the major groove and involved in hydrophobic nonstacking interactions with the base pairs along the helical major groove. This model does not require any rotation of the phenyl groups and takes into account the participation of the nonintercalated phenyl group and the other DIP ligands in supporting the binding. Furthermore, even when the model is not operating, intercalative binding of this complex could still be possible if the binding energy is large enough to overcome the barrier against rotation of the phenyl group into the plane.

#### 4.2.5. Circular Dichroism Spectroscopy

 To establish in more detail whether binding of the complexes brings about any significant conformational change of the DNA double helix, CD spectra of CT-DNA were recorded at increasing complex/CT-DNA ratios. The observed CD spectrum of natural calf thymus DNA consists of a positive band at 275 nm (UV: *k*max, 260 nm) due to base stacking and a negative band at 245 nm due to helicity, which is characteristic of DNA in right-handed B form (Figure 4) [40]. The effect of the complex on the conformation of the secondary structure of DNA was studied by keeping the concentration of CT-DNA at 5.9 × 10^−5 ^M while varying the concentration of complex in a buffer solution of 10 mM of Tris (*r*_*i*_ = 0, 0.1, 0.2, 0.4, 0.6). Spectrum of the CT-DNA and those with the additives were monitored from 215 to 800 nm. As shown in Figure 4, the CD spectrum of DNA exhibits a positive absorption at 277 nm due to the base stacking and a negative band at 240 nm due to the helicity of B-DNA. In the presence of the complex, both the positive and negative peak intensities of the CD spectra of DNA were increased. The changes in the CD spectra in the presence of the complex show stabilization of the right-handed B form of CT-DNA.

#### 4.2.6. Viscosity Measurements

**Figure 5:**
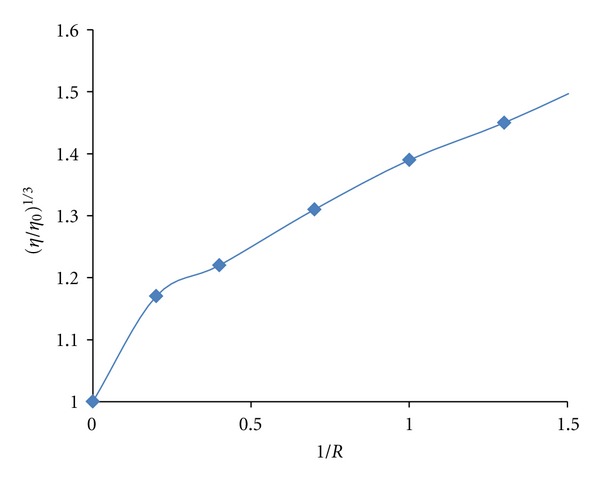
Effect of increasing amounts of Zn(II) complex on the viscosity of CT-DNA.

 The viscosity measurements of CT-DNA are regarded as the least ambiguous and the most critical tests of a binding model in solution in the absence of crystallographic structural data. A classical intercalation model demands that the DNA helix lengthens as base pairs are separated to accommodate the bound ligand, leading to the increase of DNA viscosity. In contrast, a partial, nonclassical intercalation of ligand could bend (or kink) the DNA helix, reducing its length and, concomitantly, its viscosity. In addition, complexes that bind exclusively in the DNA grooves by partial and/or nonclassical intercalation, under the same conditions, typically cause less pronounced (positive or negative) or no change in DNA solution viscosity [40, 41]. The values of relative specific viscosity (*η*/*η*_0_)^1/3^ (where *η*_0_ and *η* are the specific viscosity contributions of DNA in the absence and in the presence of the Pt complex, resp.) were plotted against 1/*R*(*R*= [DNA]/[complex] (Figure 5). It is known that the groove binder like Hoechst 33258 does not cause an increase in the axial length of the DNA [42, 43] and therefore did not alter the relative viscosity. In contrast, cisplatin which is known to kink DNA through covalent binding, shortening the axial length of the double helix [44], caused a decrease in the relative viscosity of the solution. Partial intercalators also reduce the axial length observed as a reduction in relative viscosity, whereas the classical organic intercalators such as ethidium bromide increased the axial length of the DNA and it becomes more rigid [42, 43] resulting in an increase in the relative viscosity. Results confirm the sensitivity of viscosity measurements to the different modes of DNA binding. In this study, it was observed that increasing the complex concentration leaded to an increase of the DNA viscosity. Thus we may deduce that the complex, certainly, is a DNA intercalator. Since the interaction of this complex with DNA can make DNA longer, we would expect that the relative viscosity of DNA increases with a slope between 0 and 0.96 (a value measured for ethidium bromide) [45] if the intercalation of the complex was either only one interaction mode or much stronger than other interaction(s). But, in this study, the relative viscosity of DNA increase with a slope of 0.3 (Figure 5) and it is reasonably believed that may be other interaction(s) between DNA and the complex occurred. In addition, it should be noted that the DNA binding constant measured for this complex is ten orders of magnitude lower than those determined for ethidium bromide. Therefore, the greater increase in viscosity observed for ethidium bromide compared to the Zn(II) complex is likely due to the lower binding constant of the latter to DNA. These results clearly show that the importance of using several techniques to ascertain intercalation [46].

#### 4.2.7. Electrochemical Behaviour of the Complex in the Absence and Presence of DNA

**Figure 6:**
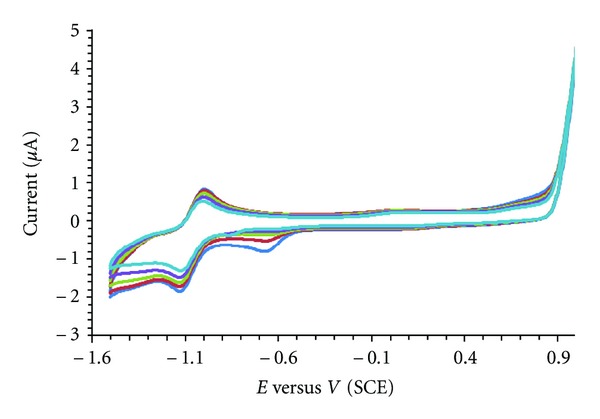
Cyclic voltammetry for the Zn(II) complex in the presence of different concentrations of CT-DNA.

 Recently, the electrochemical techniques extensively were used as a simple and rapid method to study DNA interaction with different compounds. The electrochemical behaviour of zinc is well known and was strongly influenced by the electrode material. A well-defined and sensitive peak was observed from the solutions of the complex with a GC electrode rather than the Pt one. Therefore a GC electrode was used in this investigation. When CT-DNA is added to a solution of complex both the anodic and cathodic peak current heights of the complex decreased in the same manner of increasing additions of DNA (Figure 6). Also during DNA addition the anodic peak potential (*E*_pa_), cathodic peak potential (*E*_pc_), and *E*_1/2_ (calculated as the average of *E*_pc_ and *E*_pa_) all showed positive shifts. These positive shifts are considered as evidences for intercalation of complex into the DNA, because this kind of interaction is due to hydrophobic interaction. From the other point of view, if a molecule binds electrostatically to the negatively charged deoxyribose-phosphate backbone of DNA, negative peak potential shifts should be detected. Therefore, the positive shift in the CV peak potentials of the complex is indicative of intercalative binding mode of the complex with DNA [47].

## 5. Conclusions

 In summary, we have synthesized a new tris-chelate complex of Zn(II), [Zn(DIP)_2_(DMP)](NO_3_)_2_·2H_2_O, which exhibits high binding affinity to CT-DNA. Different instrumental methods were used to finding the interaction mechanism. The following results supported the fact that the [Zn(DIP)_2_(DMP)](NO_3_)_2_·2H_2_O complex can bound to CT-DNA by the mode of partial intercalation and electrostatic binding.

1. In absorption spectrum, the absorption intensity of the complex increased (hyperchromism) evidently after the addition of DNA, which indicated the interactions between DNA and the complex.
2. The intrinsic binding constant (*K*_*b*_ = (3.1 ± 0.02) × 10^6^ M^−1^) is comparable to intercalative binding complexes.
3. Fluorescence studies showed appreciable increase in the complex emission upon addition of DNA.
4. The positive slope in van't Hoff plot indicated that the reaction of the Zn(II) complex and DNA was enthalpy favored (Δ*H* = −179.948 ± 0.6 kJ/mol).
5. The changes in the CT-CD spectra of DNA in the presence of increasing amounts of the complex show stabilization of the right-handed B form of CT-DNA.
6. The positive shift in the CV peak potentials of the complex is indicative of intercalative binding mode of the complex with DNA.
7. Increase of the relative viscosity of CT-DNA in the presence of the complex showed that the intercalative binding must be predominant.

## Figures and Tables

**Table 1:** Dynamic enhancement, bimolecular enhancement and formation constants of the complex at different temperatures.

Temperature (K)	*k*_*D*_	*k*_*B*_	*n*	*k*_*f*_ × 10^5^
298	0.343	0.343 × 10^8^	0.496	2.87
308	1.083	1.083 × 10^8^	0.484	1.97
318	1.07	1.07 × 10^8^	0.517	1.34
